# An artificial neural network to safely reduce the number of ambulance ECGs transmitted for physician assessment in a system with prehospital detection of ST elevation myocardial infarction

**DOI:** 10.1186/1757-7241-20-8

**Published:** 2012-02-01

**Authors:** Jakob L Forberg, Ardavan Khoshnood, Michael Green, Mattias Ohlsson, Jonas Björk, Stefan Jovinge, Lars Edenbrandt, Ulf Ekelund

**Affiliations:** 1 Divisions of Emergency Medicine, Department of Clinical Sciences, Skåne University Hospital at Lund, Sweden; 2 Department of Theoretical Physics, Lund University, Lund, Sweden; 3 Competence Center for Clinical Research, Skåne University Hospital at Lund, Sweden; 4 Department of Cardiology, Skåne University Hospital at Lund, Sweden; 5 Department of Clinical Physiology, Skåne University Hospital at Malmö, Sweden; 6 Department of Clinical Physiology, Sahlgrenska University Hospital, Gothenburg, Sweden

## Abstract

**Background:**

Pre-hospital electrocardiogram (ECG) transmission to an expert for interpretation and triage reduces time to acute percutaneous coronary intervention (PCI) in patients with ST elevation Myocardial Infarction (STEMI). In order to detect all STEMI patients, the ECG should be transmitted in all cases of suspected acute cardiac ischemia. The aim of this study was to examine the ability of an artificial neural network (ANN) to safely reduce the number of ECGs transmitted by identifying patients without STEMI and patients not needing acute PCI.

**Methods:**

Five hundred and sixty ambulance ECGs transmitted to the coronary care unit (CCU) in routine care were prospectively collected. The ECG interpretation by the ANN was compared with the diagnosis (STEMI or not) and the need for an acute PCI (or not) as determined from the Swedish coronary angiography and angioplasty register. The CCU physician's real time ECG interpretation (STEMI or not) and triage decision (acute PCI or not) were registered for comparison.

**Results:**

The ANN sensitivity, specificity, positive and negative predictive values for STEMI was 95%, 68%, 18% and 99%, respectively, and for a need of acute PCI it was 97%, 68%, 17% and 100%. The area under the ANN's receiver operating characteristics curve for STEMI detection was 0.93 (95% CI 0.89-0.96) and for predicting the need of acute PCI 0.94 (95% CI 0.90-0.97). If ECGs where the ANN did not identify a STEMI or a need of acute PCI were theoretically to be withheld from transmission, the number of ECGs sent to the CCU could have been reduced by 64% without missing any case with STEMI or a need of immediate PCI.

**Conclusions:**

Our ANN had an excellent ability to predict STEMI and the need of acute PCI in ambulance ECGs, and has a potential to safely reduce the number of ECG transmitted to the CCU by almost two thirds.

## Background

Reducing time to reperfusion treatment for patients with ST-segment elevation myocardial infarction (STEMI) improves patient outcomes [1-3], and every delay to primary percutaneous coronary intervention (PCI) increases long term mortality [4]. The recording of a pre-hospital 12-lead ECG in chest pain patients reduces time to PCI [5] and is an established tool to accelerate correct and timely management [6].

In order not to miss any STEMI cases, it is recommended that all ambulance-transported patients with symptoms suggesting acute coronary syndrome (ACS) have an ECG transmitted to the coronary care unit (CCU) physician on call. However, this extends the ambulance transport time in patients without STEMI [5], and could, if many ECGs are transmitted, overburden the CCU physician. A system where ECGs with a very low probability of STEMI are not transmitted would be highly useful.

Decision support tools based on artificial neural networks (ANNs) has been shown to improve junior doctors' detection of STEMI [7], to be superior to commercially available interpretation programs [8] and to be at least as good as experienced physicians to predict ACS [9] and myocardial infarction (MI) [8,10]. However, ANNs to predict STEMI have not yet been prospectively validated and compared with the real-time interpretation of CCU physicians in routine care. To our knowledge, ANNs predicting the need of acute PCI have not been presented.

The aim of this study was to examine the ability of an ANN to identify ambulance ECGs with a very low probability of STEMI and need of acute PCI, and to safely reduce the number ECGs transmitted to the CCU physician.

## Methods

### Study Population

Skåne University Hospital at Lund is a 900 bed institution with a primary catchment area of some 300 000 inhabitants, an ambulance district of about 300 000 inhabitants, and in-house PCI and coronary bypass surgery available 24 hours/day. When pre-hospital personnel suspect an ACS, a 12-lead ECG is recorded in the ambulance and electronically transmitted to the Lund CCU. The CCU physician evaluates the ECG for STEMI and decides whether or not to directly transport the patient to the PCI facility at Lund. Otherwise, the patient is transported to the nearest ED.

The CCU physician has instant access to the Region Skåne database of previously recorded ECGs and to the computerized patient records at Skåne University Hospital. The CCU physician may also in some cases call the ambulance personnel and hear a brief patient history. During the study, if the CCU physician was briefly unavailable, an experienced CCU nurse read the ECG, made the triage decision and had the decision approved by the CCU physician shortly after. Thrombolysis for STEMI was rarely, if ever, performed.

### Data collection

Between 30 August 2005 and 18 February 2006, ambulance ECGs were registered 24/7 by the CCU physicians on call at Skåne University Hospital at Lund. For each ECG the physician documented, in real-time on special forms, the identification (Y/N) of ST changes or left bundle branch block indicating a STEMI in the received ECG, as well as the decision (Y/N) to let the ambulance transport the patient directly to primary PCI. All patients deemed to have a STEMI were thus not transported directly to the PCI facility (e.g. due to terminal illness), and some patients without STEMI were transported to the PCI facility due to a suspected need of acute PCI for other causes. During the study period, a significant ST elevation was defined as an ST elevation in at least two adjacent leads ≥ 2 mm in V1-V3 and ≥ 1 mm in all other leads. The ambulance ECG was saved in the Lund ECG database.

Using the statistical software ClickView (ClickTech, Sweden), clinical data for each patient was extracted from the computerized patient records at Skåne University Hospital (Melior™, Siemens). Coronary angiography data was retrieved from the Swedish coronary angiography and angioplasty register (SCAAR) [11].

The study was approved by the regional ethics committee at Lund.

### Electrocardiography

The 12-lead ECGs in the study were recorded using computerized ECG recorders from Ortivus AB (Danderyd, Sweden) in the ambulances and Siemens-Elema AB (Solna, Sweden) in the ED. The ED ECGs (training set, see below) were traditional 12-lead ECGs with distal placement of the limb leads whereas the ambulance ECGs (testing set) had proximal placement of the limb leads ("the Lund system") [12]. This changes the waveforms slightly, but these changes have been considered clinically acceptable [12].

For the ANN, the following 13 measures were extracted from each lead: Q, R, and S amplitudes; QRS area; QRS duration; positive and negative T amplitudes; along with amplitudes of six different positions from the ST segment. In total 12 × 13 = 156 variables were created and further reduced down to 20 by principal component analysis. Reducing the number of variables used in the model in this way is warranted since there is a high degree of correlation between the measurements extracted from the 12-lead ECG. The remaining 20 variables were then normalized into Z-scores before they were used as inputs to our neural network ensemble.

### Artificial neural network ensembles

Several artificial neural networks were combined into an ensemble by bagging. The final ensemble consisted of 25 individually trained neural networks, which has been found to be sufficient in numerical studies. The ensemble prediction was calculated by averaging the outputs of its individual members. Each network consisted of a fully connected feed-forward multilayer perceptron with one hidden layer featuring 15 nodes. The networks were trained using a cross-entropy error function with an added weight elimination term that has the ability to improve generalization by controlling the complexity of the network via a tunable constant. The value of this constant, along with the number of hidden nodes, was selected through a cross-validation run on the training set. For a more general introduction to artificial neural networks see Bishop [13]. All neural networks computations were performed using in-house software.

The neural network was trained on 3000 ECGs (training set) from patients attending the ED at Skåne University hospital between 1990 and 1997 [7]. The ECGs indicating ST elevation Myocardial Infarction (STEMI) were identified by two experienced cardiologists. The ANN was only trained to detect STEMI ECG changes and not trained on coronary angiogram findings. In the present study however, the ability of the ANN to predict significant coronary artery disease on angiography was also tested (Results).

For calculation of specificity and predictive values for the ANN, the sensitivity for detecting STEMI was set to 95%. This somewhat arbitrary level was chosen in order to achieve comparable performance as with ED evaluation, where some 2-5% of the ACS patients are erroneously discharged from the ED [14,15], which implies a sensitivity of at least 95%.

### Expert consensus ECG interpretations

Two physicians highly experienced in ECG reading (UE and SJ) acted as the ECG reference standard and separately classified all 560 ECGs into: 1) ST changes/left bundle branch block as in STEMI or 2) Not STEMI. In addition to using the above mentioned ECG criteria for STEMI, these physicians also considered the configuration of the ST segment as in the clinical routine interpretation of ECGs. To somewhat mimic the situation of the CCU physician, patient records from the ambulance were available to the expert ECG interpreters. The experts made the same primary classification in 493 of the 560 ECGs. For the discrepant cases, a consensus classification was made.

### Definitions of outcomes

In this study, a STEMI was defined as a discharge diagnosis of ACS together with an ECG with ST changes/left bundle branch block as in STEMI according to the two ECG experts. The final discharge diagnose (ACS or not) was recorded from the discharge record (which included ICD10 codes) made by the ward physician and reviewed for quality by the responsible specialist ward physician, or, for patients not admitted to in-hospital care, by the responsible ED physician. The diagnostic criteria for ACS (acute myocardial infarction; AMI, or unstable angina pectoris; UA) were those recommended by the European Society of Cardiology/American College of cardiology [16] using Troponin T as the critical biomarker with a cut-off at 0.05 μg/l.

AMI was diagnosed in patients with at least one troponin T ≥ 0.05 μg/l with rising or falling on serial testing, who also had typical ischemic symptoms and/or significant ischemic ECG changes (pathological Q-wave, ST elevation, ST depression or T-wave inversion). UA was diagnosed in patients with typical ischemic symptoms with or without ischemic ECG changes and with or without slightly elevated (below AMI decision level) troponin T levels.

The need of an acute PCI was in this study defined as the patient undergoing an acute coronary angiography within 12 h after the pre-hospital ECG, with a balloon angioplasty performed due to a culprit lesion or significant coronary disease.

### Statistical analysis

Continuous variables are expressed as mean ± SD and were compared by the independent samples *t *test. Analysis for categorical variables was performed using chi-square test or Fisher exact test where appropriate. The area under the receiver-operating-characteristic curve (AUROC) was used as an overall measure of the predictive ability of the ANN. Statistical analyses were performed using SPSS 16.0.1 (SPSS Inc, Chicago, U.S.). Exact confidence intervals were calculated for sensitivity and specificity using the Clopper-Pearson method.

### Ethical approval

The Regional Ethics Committee at Lund approved the study.

## Results

### Transmitted ECGs and patient characteristics

**Figure 1:**
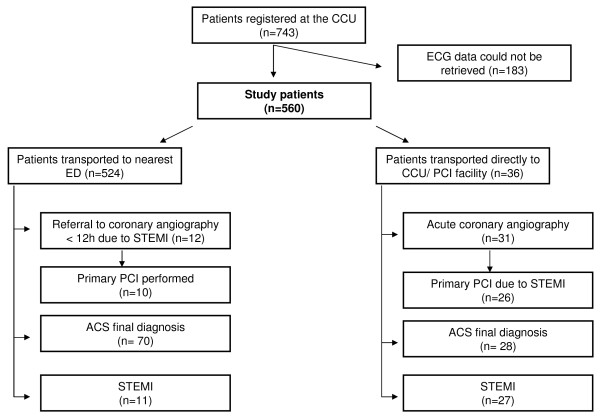
**Triage and outcome for patients with a pre-hospital ECG**.

Of the registered 743 ECGs, 560 ECGs were successfully retrieved from the local electronic ECG database and were included in the final analysis (Figure 1). Patient characteristics are given in table 1. There were no significant differences in age or ACS prevalence between the cases with missing (n = 183) and retrieved ECGs. Of the 560 ECGs, 118 were evaluated at the CCU by a specialist in cardiology, 184 by a specialist in internal medicine, 227 by a resident, and 31 initially by a CCU nurse.

**Table 1:** Patient characteristics, n = 560.

**Age**, years ± SEM	70.1 ± 0.6
**Women**	249 (45%)
**Patients admitted to in-hospital care**	417 (74%)
**Length of stay of admitted patients**, days ± SD	4.5 ± 6.2
**ACS as final diagnosis**	98 (18%)
**STEMI**	38 (7%)
**Acute coronary angiography within 12 hours due to suspicion of STEMI**	43 (8%)
**Primary PCI within 12 h from pre-hospital ECG**	36 (6%)

### Predictive performances of the ANN and the CCU physician

**Figure 2:**
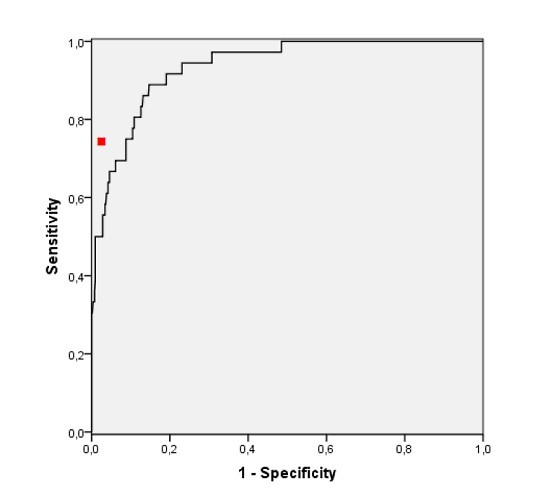
**Receiver operating characteristics curve for ANN prediction of STEMI in 560 ambulance patients with symptoms suggesting ACS**. AUROC = 0,93 (95% CI 0,89-0,96). The red dot indicates the performance of the CCU physician in predicting STEMI.

**Figure 3:**
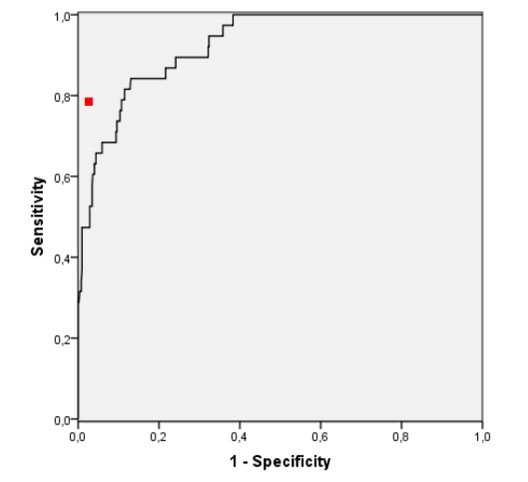
**Receiver operating characteristic curve for ANN prediction of the need of acute PCI within 12 hours in 560 patients with symptoms suggesting ACS**. AUROC = 0,94 (95% CI 0,90-0,97). The red dot indicates the performance of the CCU physician in predicting the need of acute PCI.

The predictive performances of the ANN as compared to the CCU physician are given in Table 2. If the sensitivity of the ANN was set to the same level as the CCU physician (0.74) the specificity of the ANN for STEMI and a need of acute PCI was 0.90 (95% CI 87-93%) and 0.90 (95% CI 87-93%), respectively. The AUROC of the ANN was 0.93 (95% CI 0.89-0.96; Figure 2) for the ability to detect STEMI, and 0.94 (95% CI 0.90-0.97; Figure 3) for predicting the need of acute PCI.

**Table 2:** Predictive performances of the CCU physician and the ANN.

	Sens	Spec	PPV	NPV
**Predicting STEMI**				
ANN	0,95 (0,82-0,99)	0,68 (0,63-0,73)	0,18 (0,13-0,23)	0,99 (0,98-1,00)
CCU physician	0,74 (0,57-0,87)	0,98 (0,97-0,99)	0,76 (0,59-0,088)	0,98 (0,97-1,00)
ANN and CCU physician*	0,74 (0,57-0,87)	0,99 (0,98-1,0)	0,80 (0,63-0,92)	0,98 (0,97-0,99)
**Predicting need of acute PCI**				
ANN	0,97 (0,85-1,0)	0,68 (0,63-0,72)	0,17 (0,12-0,23)	1,0 (0,98-1,00)
CCU physician	0,78 (0,61-0,90)	0,98 (0,97-0,99)	0,76 (0,59-0,89)	0,98 (0,97-0,99)
ANN and CCU physician*	0,78 (0,61-0,90)	0,99 (0,98-1,0)	0,80 (0,63-0,92)	0,98 (0,97-0,99)

Sens; sensitivity, Spec; specificity, PPV; positive predictive value, NPV; negative predictive value *Theoretical diagnostic performances if only ECGs in which the ANN predicted STEMI were to be transmitted to the CCU physician.

When the ANN was set to 95% (95% CI 82-99%) sensitivity for STEMI, it had 97% (95% CI 85-100%) sensitivity for the need for acute PCI.

### Patients with STEMI or a need of acute PCI not identified by the ANN

With the STEMI sensitivity set to 95%, the ANN missed two patients with STEMI. None of these patients had ECGs that were classified as STEMI by the CCU physician, and none underwent an acute PCI.

With this ANN setting, the ANN missed one patient without STEMI who needed an acute PCI. This patient was also missed by the CCU physician, i.e. the patient was triaged to the ED. Due to progressing ECG changes the patient underwent an acute PCI 3 h and 48 min after the prehospital ECG recording.

### Patients identified correctly only by the ANN

Eight patients with STEMI were correctly identified by the ANN, but not classified as STEMI by the CCU physician.

In eight patients with a need of acute PCI, the CCU physician neither referred the patient to an acute PCI nor classified the patients as having a STEMI. Only one of these patients had a STEMI according to the definition in this study, but, interestingly, seven of these patients were identified by the ANN.

### Effects of ANN screening before ECG transmission to the CCU

If ambulance ECGs where the ANN did not identify a STEMI or a need of acute PCI were theoretically to be withheld from transmission, the number of ECGs evaluated in the CCU could have been reduced from 560 to 204 (by 64%) without missing any case with STEMI or a need of immediate PCI. The serially combined predictive performance of the ANN and the CCU physician is shown in Table 2.

## Discussion

In this study, we present an ANN with the ability to predict STEMI and a true need for acute PCI in prehospital chest pain patients. The large ANN AUROCs (0.93 and 0.94 for detecting STEMI and need of acute PCI) imply an excellent predictive ability, which could potentially be used to safely reduce the number of ambulance ECGs sent to the CCU by almost two thirds. To our knowledge, the present study is the first to demonstrate a decision support tool that can predict the presence of a culprit lesion or significant coronary artery disease.

At a STEMI sensitivity of 95%, the sensitivity of our ANN for a need of acute PCI was as high as 97% which was clearly better than the 74% sensitivity of the CCU physician. Among eight patients with a need of acute PCI missed by the CCU physician, the ANN correctly identified seven. However, the specificity of the ANN was only 68%, and the positive predictive value (PPV) in our population only 18%.

Should the ANN be implemented in a prehospital ECG system, a final ECG interpretation and triage decision by a physician is therefore clearly needed to avoid unnecessary catheterization laboratory activation. Pain history and symptoms suggesting other causes (e.g. aortic dissection) of chest pain, co-morbidities and CCU bed/PCI availability are usually important information for the final triage decision, and a high specificity in the triage decision is also needed because coronary angiographies carry a risk of complications. The low PPV of the ANN was of course also related to the STEMI prevalence of only 7%, which is lower than reported by Sejersten et al. (28%) [5] and Clark et al. (34%) [17]. This indicates a very low threshold among the ambulance personnel to transmit an ECG. The STEMI prevalence in our material was in fact comparable to the estimated real prevalence in the prehospital setting [18].

In Table 2, the serially combined predictive performance of the ANN and the CCU physician is shown. These data are based on a system where the decisions of the CCU physician are not influenced by the ANN. As output, our ANN generates a likelihood of STEMI/need of acute PCI. Should this information be available to the CCU physician, we believe it is likely that the serially combined performance would improve towards the higher sensitivity of the ANN. If so, the ANN would enable the physician to miss fewer patients with STEMI and a need of acute PCI. In this way, eight of the 36 patients (22%) in need of acute PCI that was not detected by the CCU physician could theoretically have had the time to reperfusion reduced if the ANN results were available to the CCU physician. The true effects of our ANN in such a system remain to be established.

Methods to safely reduce the number of ECGs transmitted to the CCU could include ECG interpretation by 1) the ambulance personnel, 2) computer programs with rule-based interpretation or 3) ANN computer programs. Studies indicate that ambulance paramedics can reliably interpret the ECG [19], but this requires additional training for all personnel. Also, the number of false negative cases in routine care has to our knowledge not been reported. Studies of rule-based ECG interpretation programs in routine care have shown a sensitivity and specificity for STEMI of 78% and 91-94% [17] respectively, thus missing about 1 in 5 STEMI cases. When our ANN was set to 95% sensitivity it did not miss any STEMI case that was detected by the CCU physician.

When setting the ANN to the sensitivity of the CCU physician, the ANN specificity for STEMI was lower (0.91 vs 0.98). In this prospective routine care study, and at the sensitivity of the CCU physician, we could thus not confirm the reported superior STEMI prediction by an ANN compared to physicians in retrospective ECGs [8]. There might be at least two reasons for this. First, the CCU physician had access to previous ECGs, medical records and a brief clinical history, which should have improved specificity when predicting STEMI. Evaluating also previous ECGs have been shown to improve physicians' specificity for STEMI and to reduce CCU admissions [20]. Feeding also a previous ECG to the ANN, if technically feasible, could be a way of improving the ANN's specificity. In a previous study, the ability of an ANN to predict AMI was significantly improved when a previous ECG was supplied together with the new ECG [21]. The benefit of previous ECGs should even be larger when predicting STEMI than when predicting AMI, since ECG changes are less specific in the average AMI case. Secondly, the ANN was not trained on prehospital ECGs. In addition to the slightly different ECG appearance with the prehospital lead placement, the performance of our ANN could be reduced by the sometimes poorer technical quality of the pre-hospital ECG compared to the in-hospital ECGs in the training set.

The clearly higher specificity than sensitivity of the CCU physicians in routine care in this and other studies [5,17] may seem surprising, but should be viewed in the context of a constant bed shortage in the CCU and high catheterization laboratory utilization, which forces the physicians to focus on specificity more than sensitivity. The reasoning is that the patient can always be secondarily transferred from the ED to the CCU of to acute PCI.

Among the present ambulance patients, 98 (18%) had ACS as the final discharge diagnosis. ANNs have been shown to be superior to experienced cardiologists to predict both MI and ACS [8]. In the prehospital setting, an ANN could therefore not only be used to predict STEMI and a need of PCI, but also ACS. This could potentially improve the pre-hospital triage of chest pain patients and contribute to a more timely ACS treatment, perhaps especially if cardiac biomarkers in the ambulance were also analyzed [22].

### Clinical implications

If the high diagnostic performance of our ANN is confirmed in other cohorts, we believe that it could be used to reduce the number of ambulance ECG's transmitted for expert interpretation in patients with symptoms suggesting ACS. The ANN interpretation will be available instantly, and if STEMI is not detected, transport to the nearest ED could be initiated without delay. It is even possible that a fast and easy ANN interpretation would increase the number of prehospital ECGs registered, and increase the number of STEMI patients identified already in the ambulance. If the ANN suggests a STEMI or a need of acute PCI, the ECG should be transmitted to a cardiologist for interpretation and a final triage decision. If introduced, there is of course a risk that ECG interpretation skills could decrease among EMS staff. However, future ANNs could perhaps explain their interpretations and instead help educate the EMS staff [23].

### Limitations

The ANN was evaluated in only one pre-hospital district, and the predictive performance of the ANN and the potential reduction in the ECGs sent to the CCU are therefore not necessarily generalizable to other districts. This ANN should not be clinically applied outside our district before validation in new cohorts.

We were unable to retrieve 183 ECGs due to 1) technical problems when trying to save the ECG, 2) ECG could not be saved because the patient was not a Swedish citizen or not reliably identified, or 3) the CCU nurse did not save the ECG to the database. The ACS prevalence was not significantly different among patients with and without saved ECGs, and it is unlikely that missing these ECGs had any significant effect on the results.

No follow-up of patients discharged from the ED was performed in this study. Some patients with STEMI and a need of PCI might therefore have been missed both by the CCU physician and the ED physician, and discharged home from the ED. Although we cannot completely exclude this possibility, we consider it highly unlikely since the ED physician had access to both the prehospital and the ED ECG.

## Conclusions

In the present study, we demonstrate for the first time an ANN with an ability to predict a true need for an acute PCI. The AUROC was large indicating an excellent overall predictive performance. Set to a high sensitivity, the ANN could be used to identify patients with a very low likelihood of a STEMI or a need of acute PCI, where the ECG does not need to be sent to the CCU physician for assessment. Using the ANN in this way could potentially reduce the number of ECG transmitted to the CCU by almost two thirds without missing any patient needing an acute PCI.

## Competing interests

The authors declare that they have no competing interests.

## Authors' contributions

JLF participated in the design of the study, data acquisition, data analysis, and wrote the manuscript. AK collected and analysed data. MG and MO constructed the ANN model. JB participated in the statistical analysis. SJ made the expert ECG interpretations. LE participated in the design of the study. UE participated in the conception and design of the study, expert ECG interpretation, managed the project and wrote the manuscript. All authors have contributed with critical revisions of the manuscript and have read and approved the final version.

## References

[B1] De Luca G Suryapranata H Ottervanger JP Antman EM Time delay to treatment and mortality in primary angioplasty for acute myocardial infarction: every minute of delay counts Circulation 2004 109 10 1223 1225 10.1161/01.CIR.0000121424.76486.20 15007008

[B2] McNamara RL Wang Y Herrin J Curtis JP Bradley EH Magid DJ Peterson ED Blaney M Frederick PD Krumholz HM NRMI Investigators Effect of door-to-balloon time on mortality in patients with ST-segment elevation myocardial infarction J Am Coll Cardiol 2006 47 11 2180 2186 10.1016/j.jacc.2005.12.072 16750682

[B3] Cannon CP Gibson CM Lambrew CT Shoultz DA Levy D French WJ Gore JM Weaver WD Rogers WJ Tiefenbrunn AJ Relationship of symptom-onset-to-balloon time and door-to-balloon time with mortality in patients undergoing angioplasty for acute myocardial infarction JAMA 2000 283 22 2941 2947 10.1001/jama.283.22.2941 10865271

[B4] Terkelsen CJ Sorensen JT Maeng M Jensen LO Tilsted HH Trautner S Vach W Johnsen SP Thuesen L Lassen JF System delay and mortality among patients with STEMI treated with primary percutaneous coronary intervention JAMA 2010 304 7 763 771 10.1001/jama.2010.1139 20716739

[B5] Sejersten M Sillesen M Hansen PR Nielsen SL Nielsen H Trautner S Hampton D Wagner GS Clemmensen P Effect on treatment delay of prehospital teletransmission of 12-lead electrocardiogram to a cardiologist for immediate triage and direct referral of patients with ST-segment elevation acute myocardial infarction to primary percutaneous coronary intervention Am J Cardiol 2008 101 7 941 946 10.1016/j.amjcard.2007.11.038 18359312

[B6] Bradley EH Nallamothu BK Curtis JP Webster TR Magid DJ Granger CB Moscucci M Krumholz HM Summary of evidence regarding hospital strategies to reduce door-to-balloon times for patients with ST-segment elevation myocardial infarction undergoing primary percutaneous coronary intervention Crit Pathw Cardiol 2007 6 3 91 97 10.1097/HPC.0b013e31812da7bc 17804968

[B7] Olsson SE Ohlsson M Ohlin H Dzaferagic S Nilsson ML Sandkull P Edenbrandt L Decision support for the initial triage of patients with acute coronary syndromes Clin Physiol Funct Imaging 2006 26 3 151 156 10.1111/j.1475-097X.2006.00669.x 16640509

[B8] Heden B Ohlin H Rittner R Edenbrandt L Acute myocardial infarction detected in the 12-lead ECG by artificial neural networks Circulation 1997 96 6 1798 1802 10.1161/01.cir.96.6.1798 9323064

[B9] Forberg JL Green M Bjork J Ohlsson M Edenbrandt L Ohlin H Ekelund U In search of the best method to predict acute coronary syndrome using only the electrocardiogram from the emergency department J Electrocardiol 2009 42 1 58 63 10.1016/j.jelectrocard.2008.07.010 18804783

[B10] Xue J Aufderheide T Scott Wright R Klein J Farrell R Rowlandson I Young B Added value of new acute coronary syndrome computer algorithm for interpretation of prehospital electrocardiograms J Electrocardiol 2004 37 Suppl 233 239 10.1016/j.jelectrocard.2004.08.063 15534847

[B11] Swedish Angiography and Angioplasty Registry http://www.ucr.uu.se/scaar/

[B12] Pahlm O Wagner GS Proximal placement of limb electrodes: a potential solution for acquiring standard electrocardiogram waveforms from monitoring electrode positions J Electrocardiol 2008 41 6 454 457 10.1016/j.jelectrocard.2008.06.019 18805543

[B13] Bishop CM Neural Networks for Pattern Recognition: 1995 Oxford University Press

[B14] Lee TH Rouan GW Weisberg MC Brand DA Acampora D Stasiulewicz C Walshon J Terranova G Gottlieb L Goldstein-Wayne B Clinical characteristics and natural history of patients with acute myocardial infarction sent home from the emergency room Am J Cardiol 1987 60 4 219 224 10.1016/0002-9149(87)90217-7 3618483

[B15] Pope JH Aufderheide TP Ruthazer R Woolard RH Feldman JA Beshansky JR Griffith JL Selker HP Missed diagnoses of acute cardiac ischemia in the emergency department N Engl J Med 2000 342 16 1163 1170 10.1056/NEJM200004203421603 10770981

[B16] Alpert JS Thygesen K Antman E Bassand JP Myocardial infarction redefined--a consensus document of The Joint European Society of Cardiology/American College of Cardiology Committee for the redefinition of myocardial infarction J Am Coll Cardiol 2000 36 3 959 969 10.1016/s0735-1097(00)00804-4 10987628

[B17] Clark EN Sejersten M Clemmensen P Macfarlane PW Automated electrocardiogram interpretation programs versus cardiologists' triage decision making based on teletransmitted data in patients with suspected acute coronary syndrome Am J Cardiol 2010 106 12 1696 1702 10.1016/j.amjcard.2010.07.047 21126612

[B18] Youngquist ST Kaji AH Lipsky AM Koenig WJ Niemann JT A Bayesian sensitivity analysis of out-of-hospital 12-lead electrocardiograms: implications for regionalization of cardiac care Acad Emerg Med 2007 14 12 1165 1171 10.1197/j.aem.2007.07.009 18045892

[B19] Whitbread M Leah V Bell T Coats TJ Recognition of ST elevation by paramedics Emerg Med J 2002 19 1 66 67 10.1136/emj.19.1.66 PMC1725764 11777883

[B20] Lee TH Cook EF Weisberg MC Rouan GW Brand DA Goldman L Impact of the availability of a prior electrocardiogram on the triage of the patient with acute chest pain J Gen Intern Med 1990 5 5 381 388 10.1007/BF02599421 2231032

[B21] Ohlsson M Ohlin H Wallerstedt SM Edenbrandt L Usefulness of serial electrocardiograms for diagnosis of acute myocardial infarction Am J Cardiol 2001 88 5 478 481 10.1016/s0002-9149(01)01722-2 11524053

[B22] Sorensen JT Terkelsen CJ Steengaard C Lassen JF Trautner S Christensen EF Nielsen TT Botker HE Andersen HR Thygesen K Prehospital troponin T testing in the diagnosis and triage of patients with suspected acute myocardial infarction Am J Cardiol 2011 107 10 1436 1440 10.1016/j.amjcard.2011.01.014 21414596

[B23] Green M Ekelund U Edenbrandt L Bjork J Forberg JL Ohlsson M Exploring new possibilities for case-based explanation of artificial neural network ensembles Neural Netw 2009 22 1 75 81 10.1016/j.neunet.2008.09.014 19038532

